# Effect of Erosive Agents on Surface Characteristics of Nano-Fluorapatite Ceramic: An In-Vitro Study

**DOI:** 10.3390/molecules27154691

**Published:** 2022-07-22

**Authors:** Navara Tanweer, Fazal-Ur-Rehman Qazi, Gotam Das, Afreen Bilgrami, Sakeenabi Basha, Naseer Ahmed, Hammam Ahmed Bahammam, Sarah Ahmed Bahammam, Syed Nahid Basheer, Ali A. Assiry, Mohmed Isaqali Karobari, Abdul Samad Khan, Artak Heboyan

**Affiliations:** 1 Department of Dental Materials, Sir Syed Dental College, Karachi 75600, Pakistan; drnavarakhan@gmail.com; 2 Department of Operative Dentistry, Dr. Ishrat-Ul-Ebad Khan Institute of Oral Health Sciences, Dow University of Health Sciences, Karachi 74200, Pakistan; qazirehman@hotmail.com; 3 Department of Prosthodontics, College of Dentistry, King Khalid University, Abha 62529, Saudi Arabia; gmenghwar@kku.edu.sa; 4 Department of Dental Materials, Fatimah Jinnah Dental College, Karachi 74900, Pakistan; afreenagha@hotmail.com; 5 Department of Community Dentistry, Faculty of Dentistry, Taif University, Taif, 21944, Saudi Arabia; sakeena@tudent.edu.sa; 6 Prosthodontics Unit, School of Dental Sciences, Health Campus, Universiti Sains Malaysia, Kota Bharu 16150, Malaysia; naseerahmed@student.usm.my; 7 Department of Prosthodontics, Altamash Institute of Dental Medicine, Karachi 75500, Pakistan; 8 Department of Pediatric Dentistry, College of Dentistry, King Abdulaziz University, Jeddah 80290, Saudi Arabia; habahammam@kau.edu.sa; 9 Department of Pediatric Dentistry and Orthodontics, College of Dentistry, Taibah University, Medina 42353, Saudi Arabia; sbahammam@taibahu.edu.sa; 10 Department of Restorative Dental Sciences, College of Dentistry, Jazan University, Jazan 45142, Saudi Arabia; syednahidbasheer@gmail.com; 11 Preventive Dental Science Department, Faculty of Dentistry, Najran University, Najran 55461, Saudi Arabia; 12 Department of Conservative Dentistry & Endodontics, Saveetha Dental College & Hospitals, Saveetha Institute of Medical and Technical Sciences University, Chennai 600077, Tamil Nadu, India; 13 Department of Restorative Dentistry & Endodontics, Faculty of Dentistry, University of Puthisastra, Phnom Penh 12211, Cambodia; 14 Department of Restorative Dental Sciences, College of Dentistry, Imam Abdulrahman Bin Faisal University, Dammam 31441, Saudi Arabia; akhan@iau.edu.sa; 15 Department of Prosthodontics, Faculty of Stomatology, Yerevan State Medical University after Mkhitar Heratsi, Str. Koryun 2, Yerevan 0025, Armenia

**Keywords:** dental ceramic, erosive, beverages, surface roughness, surface topography

## Abstract

Erosive beverages cause dissolution of natural teeth and intra-oral restorations, resulting in surface characteristic changes, particularly roughness and degradation. The purpose of this study was to evaluate the surface roughness and topography of a dental ceramic following immersion in locally available erosive solutions. A total of 160 disc specimens of a nano-fluorapatite type ceramic (12 mm diameter and 2 mm thickness) were fabricated and equally distributed into two groups (*n* = 80) and then evenly distributed among the following five testing groups (*n* = 16): lemon juice, citrate buffer solution, 4% acetic acid, soft cola drink, and distilled water which served as a control. The surface roughness (Ra) and topography were evaluated using a profilometer and scanning electron microscope at baseline, 24 h, 96 h, and 168 h respectively. Data were analyzed using ANOVA and Tukey’s multiple comparisons (*p* ≤ 0.05). Surface changes were observed upon exposure to all acidic beverages except distilled water. Amongst all immersion media, 4% acetic acid produced the most severe surface roughness across all time periods (i.e., baseline, 24 h, 96 h, and 168 h). A statistically significant difference in the surface roughness values between all immersion media and across all four time intervals was observed. Erosive agents had a negative effect on the surface roughness and topography of the tested ceramic. The surface roughness increased with increased storage time intervals.

## 1. Introduction

The oral cavity is one of the harshest environments of the human body. Hence, teeth and dental restorations, if present; are regularly subjected to food items that produce large variations in the oral pH and temperature [1]. Therefore, it is highly recommended that the material employed for restoring teeth should be capable enough of withstanding such intraoral changes.

Ceramic materials have long been admired, not only for their pleasing esthetic qualities [2] but also for their wear resistance, biocompatibility, and inertness, making them suitable for veneers, fixed prosthesis, and inlay/onlays [3,4]. Although dental ceramics are considered to be chemically inert, their chemical stability is influenced by the elemental composition, microstructure, and chemical character of the erosive agent, and changes in oral temperature and exposure time [5]. Varieties of ceramic systems have been introduced in clinical dentistry such as feldspathic porcelain, aluminosilicate glasses, lithium disilicate, and leucite-based ceramics. A recent all-ceramic nano-fluorapatite ceramic has been introduced, which is composed of glass ceramic (SiO_2_–LiO_2_–Na_2_O–K_2_O–Al_2_O_3_–CaO) and crystals of fluorapatite; these Ca_5_(PO_4_)_3_FV crystals of fluorapatite (Ca_10_(PO_4_)_6_F_2_ are dispersed in a feldspathic glass matrix, making the microstructure more refined than any other available ceramic system found commercially [6].

Literature shows that the intake of fruit juices and cola drinks has lately been increasing [7,8]. When dental ceramic restorations are exposed to such erosive beverages they produce surface degradation, subsequently leading to crack propagation within the ceramic structure [6,9]. This phenomenon is believed to be a result of leaching out of the alkali ions, which tend to be less stable in the glass phase in comparison to the crystalline phase. As a consequence of such degradation, the exposed ceramic surface will eventually be roughened, thereby promoting greater plaque accumulation, discoloration, and weakening of ceramic structure as well as resulting in the wearing of antagonist natural teeth and restorations [10,11,12,13,14]. Kukiattrakoon et al. [1] reported a significant change in surface roughness values of different dental ceramic materials when exposed to different erosive agents. It was also observed that surface roughness values of fluorapatite-type porcelain were not significantly different, except when immersed in 4% acetic acid. A similar study revealed that storage of ceramic material in different acidic mediums resulted in an increase in their surface roughness in comparison to when they were stored in a control (deionized water) medium [15]. Kukiattrakoon et al. [16], in another study, evaluated the effect of acidic agents on a range of dental porcelain utilizing a scanning electron microscope (SEM) and reported that erosive agents produced surface destruction of all ceramic types used. Another study investigated the effect of carbonated drinks on two ceramic materials using SEM analysis, and concluded that Coca-Cola produced an etched surface on dental ceramics along with significant surface roughness changes [17].

Literature regarding the changes in surface topography, micro-hardness and flexural strength, as well as the elemental release of various ceramic systems following exposure to erosive agents, is currently available [18,19,20,21]. However, to the best of our knowledge, scientific literature to assess the surface roughness of newly developed nano-fluorapatite type dental ceramics is limited. Hence, the purpose of this in-vitro study was to measure the surface roughness changes and topography of IPS e.max Ceram (a nano-fluorapatite type ceramic) upon immersion in erosive agents (lemon juice, citrate buffer solution, 4% acetic acid and a carbonated drink).

## 2. Results

### 2.1. Mean Surface Roughness in Terms of Medium I—Distilled Water

In terms of immersion in distilled water, the overall mean surface roughness values (as per profilometer readings) of ceramic discs at baseline, 24 h, 96 h and 168 h were found to be 0.2238 (±0.0048) Ra, 0.2240 (±0.0051) Ra, 0.2241 (±0.0052) Ra and 0.2245 (±0.0057) Ra, respectively (Table 1). The results show that there was a very minor change in the surface roughness of ceramic disc specimens when immersed in distilled water for different time periods.

### 2.2. Mean Surface Roughness in Terms of MEDIUM III—Citrate Buffer

**Figure 1:**
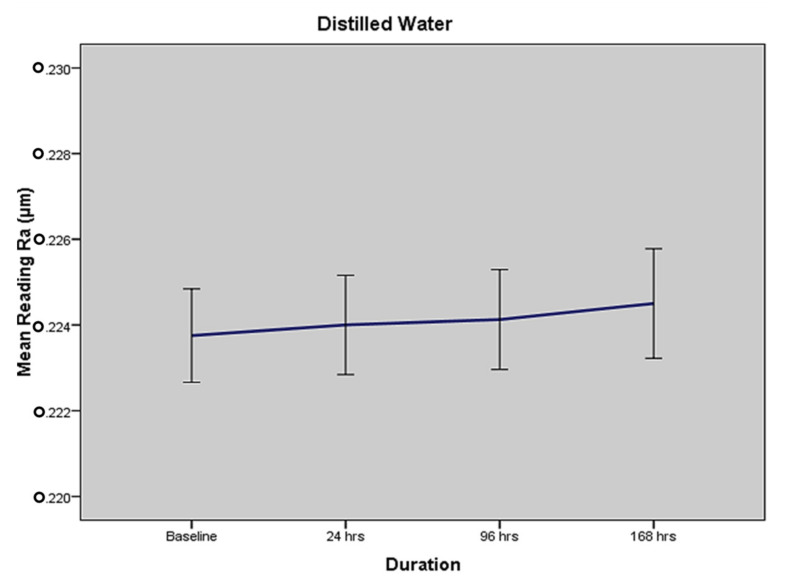
Time related change in surface roughness (mean Ra with standard error) for distilled water.

**Figure 2:**
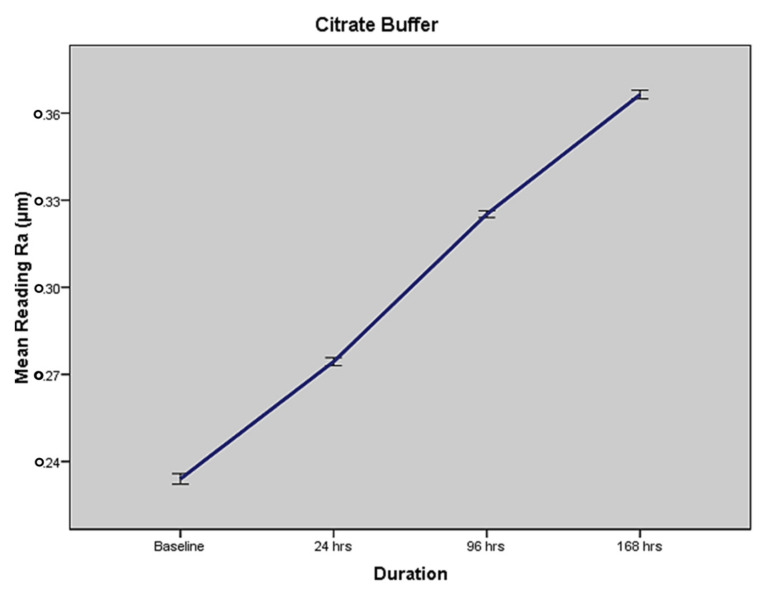
Time related change in surface roughness (mean Ra with standard error) for citrate buffer.

In terms of immersion in citrate buffer, the overall mean surface roughness values (as per profilometer readings) of ceramic discs at baseline, 24 h, 96 h and 168 h were found to be 0.2238 (±0.0048) Ra, 0.2744 (±0.0059) Ra, 0.3252 (±0.0050) Ra and 0.3664 (±0.0065) Ra, respectively, as shown in Table 2. The results show a time-dependent increase in the surface roughness and degradation of ceramic disc specimens when immersed in the citrate buffer solution. It produced the second-highest surface roughness on the disc specimens. Figure 1 and Figure 2 show the individual results for distilled water and the citrate buffer. The other media, i.e., lemon juice, acetic acid, and cola, have individual results and exhibited a time-dependent increase in surface roughness.

### 2.3. Comparison of Surface Roughness Assessment at Different Time Intervals for Specified Immersion Solutions

**Figure 3:**
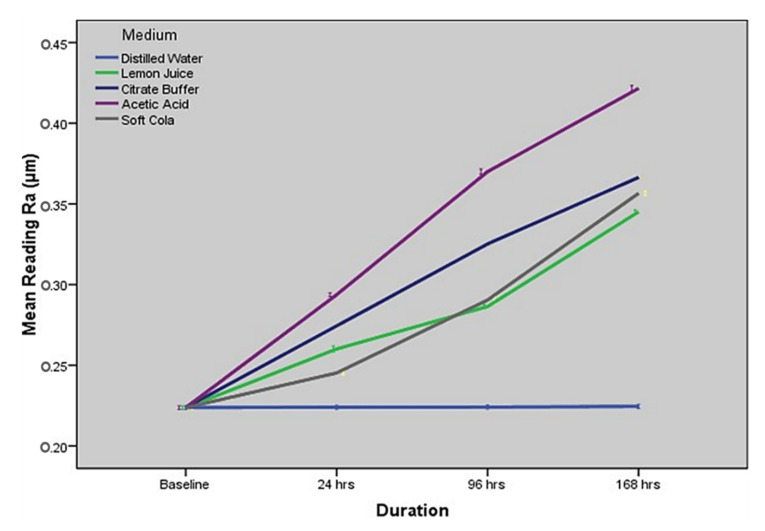
Comparison of surface roughness (mean Ra with standard error) across four different time intervals for each of the five immersion solution.

Figure 3 and Table 3 show the comparison (based on one-way ANOVA) of surface roughness values of nano-fluorapatite ceramic at baseline, 24, 96 and 168 h for every tested immersion solution. The comparison shows statistically significant differences in all immersion media except distilled water across all four time intervals.

For the distilled water group, one-way ANOVA indicated a statistically non-significant difference among surface roughness at baseline, 24 h, 96 h and 168 h (*p* < 0.8289). For the lemon juice group, it showed a statistically significant difference between each time period with regards to surface roughness (*p* < 0.0001 for all comparisons as generated by one-way ANOVA and post hoc Tukey’s HSD). Similar statistically significant differences in surface roughness across each time period were also evident in the citrate buffer (*p* < 0.0001), 4% acetic acid (*p* < 0.0001), and the soft cola drink (*p* < 0.0001) groups.

### 2.4. Comparison of Surface Roughness amongst Immersion Solutions at Specified Time Intervals

Table 4 shows a comparison of surface roughness of nano-fluorapatite ceramic amongst the five immersion media at different time intervals. The results show a statistically significant difference in the surface roughness values of ceramic disc specimens between the five immersion media at every specific time period (i.e., 24, 96, and 168 h, respectively).

### 2.5. Surface Topography Analysis Using SEM (Non-Contact Method)

**Figure 4:**
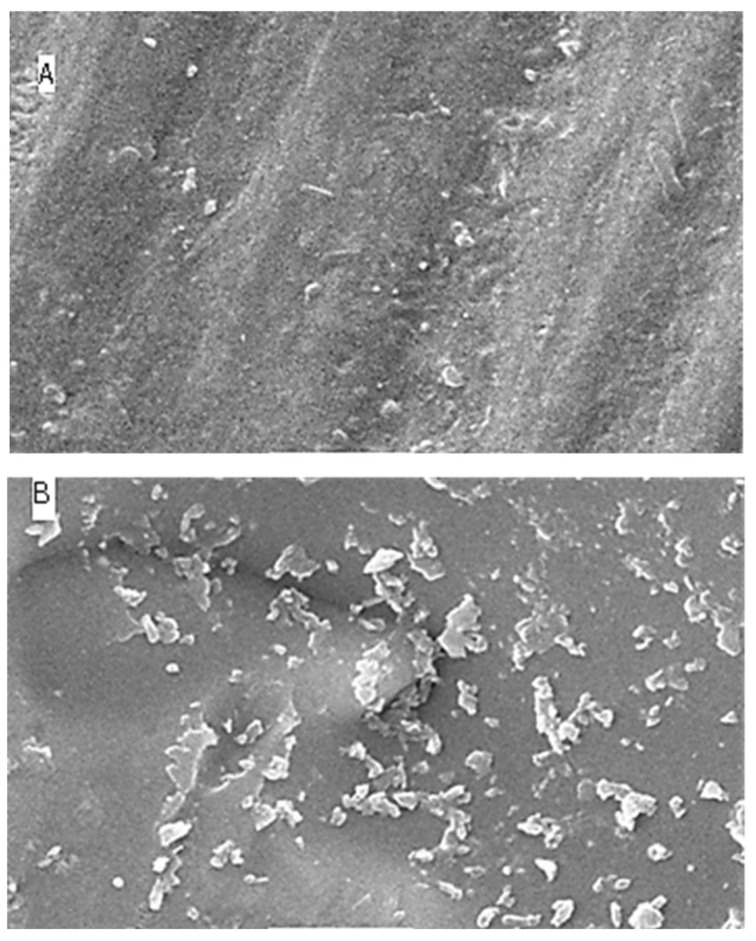

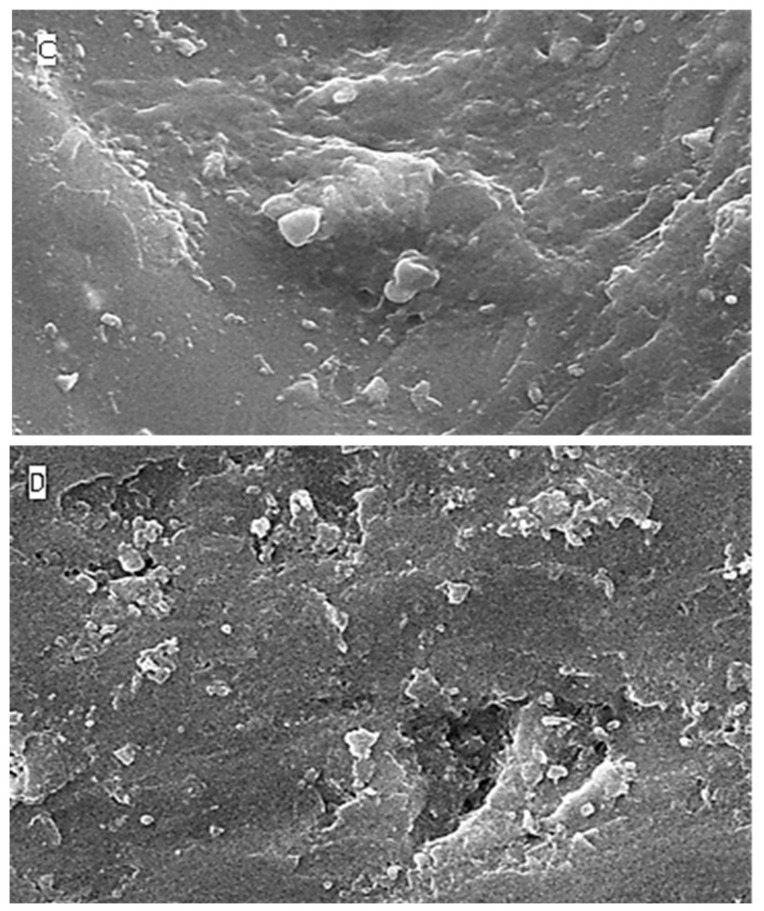
SEM images (**A**–**D**) of surface roughness at baseline, 24 h, 96 h and 168 h in distilled water. (**A**): Baseline SEM photomicrograph of IPS e.max ceramic before immersion in DW (×5000). (**B**): SEM photomicrograph of IPS e.max ceramic After 24 h immersion in DW (×5000). (**C**): SEM photomicrograph of IPS e.max ceramic After 96 h immersion in DW (×5000). (**D**): SEM photomicrograph of IPS e.max ceramic After 168 h immersion in DW (×5000).

**Figure 5:**
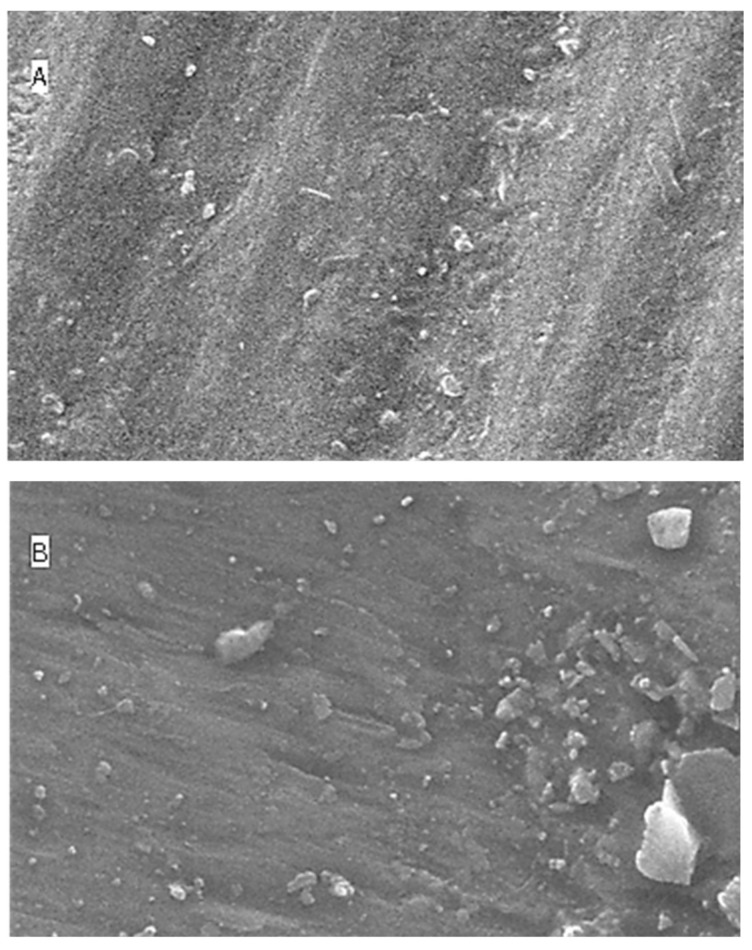

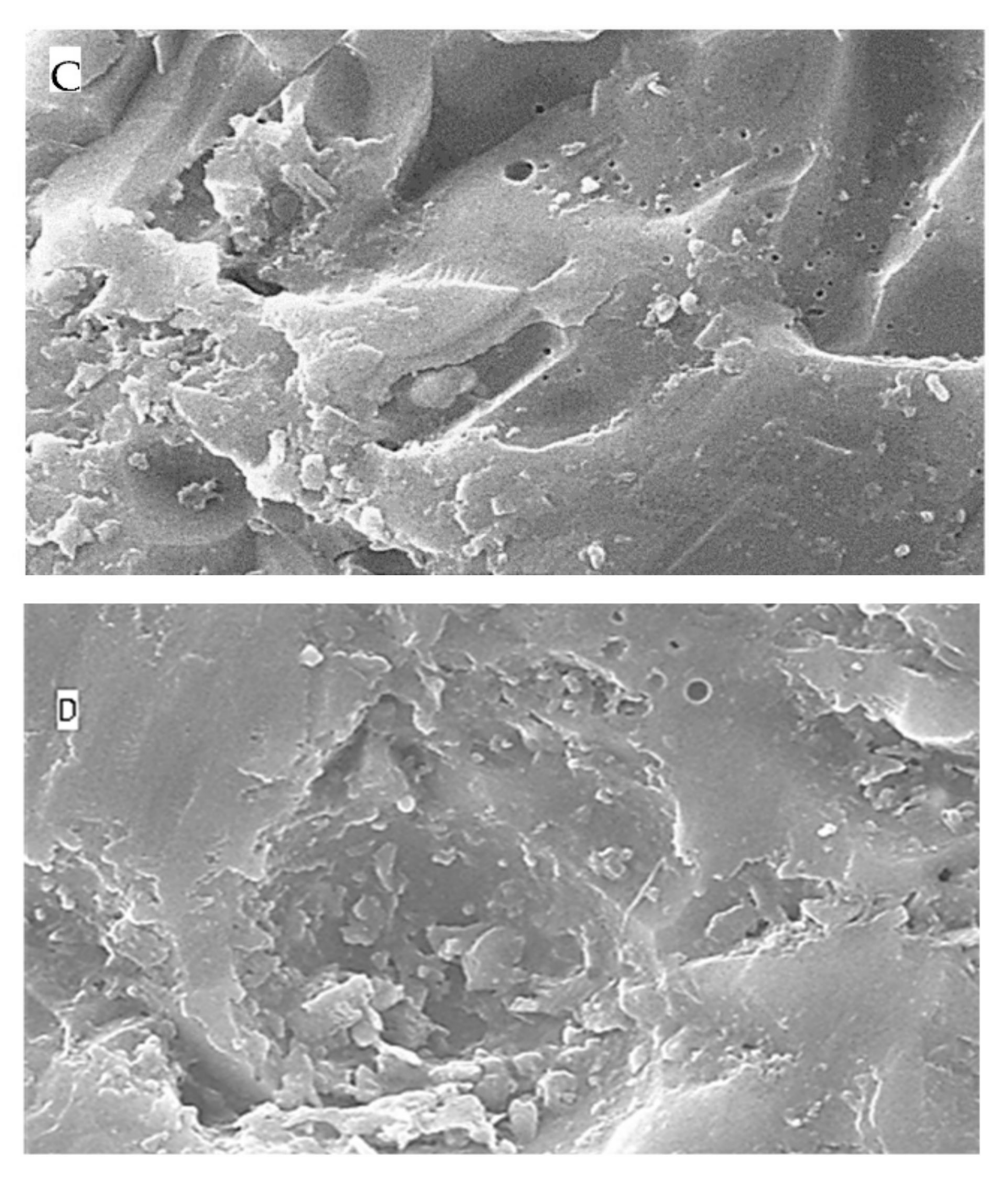
SEM images (**A**–**D**) of surface roughness at baseline, 24 h, 96 h and 168 h in lemon juice. (**A**): Baseline SEM photomicrograph of IPS e.max ceramic before immersion in lemon juice (×5000). (**B**): SEM photomicrograph of IPS e.max ceramic after 24 h immersion in lemon juice (×5000). (**C**): SEM photomicrograph of IPS e.max ceramic after 96 h immersion in lemon juice (×5000). (**D**): SEM photomicrograph of IPS e.max ceramic after 168 h immersion in lemon juice (×5000).

**Figure 6:**
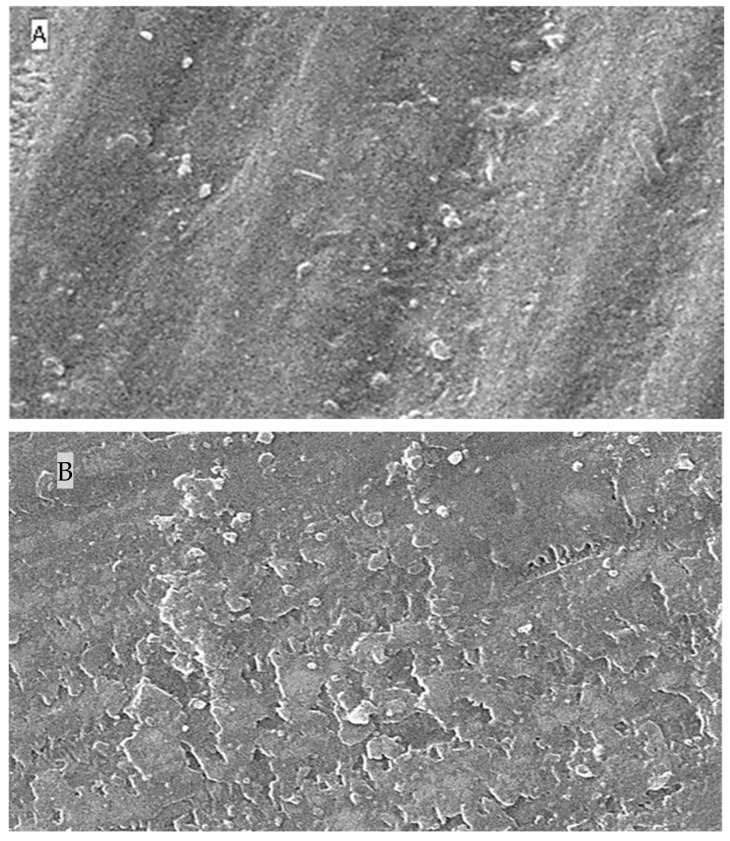

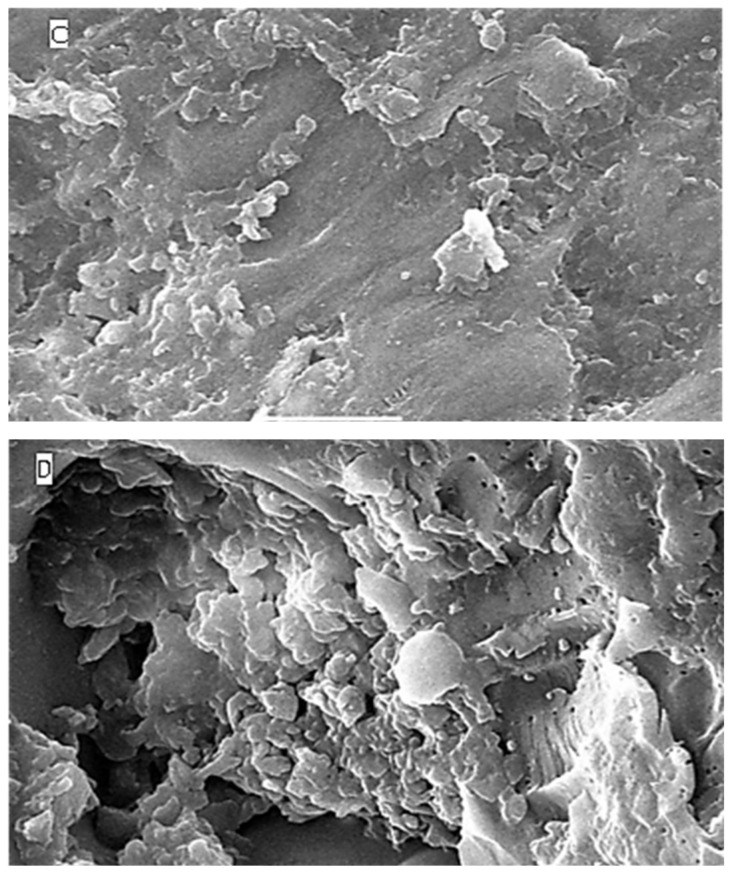
SEM images (**A**–**D**) of surface roughness at baseline, 24 h, 96 h and 168 h in citrate buffer. (**A**): Baseline SEM photomicrograph of IPS e.max ceramic before immersion in citrate buffer (×5000). (**B**): SEM photomicrograph of IPS e.max ceramic after 24 h immersion in citrate buffer (×5000). (**C**): SEM photomicrograph of IPS e.max ceramic After 96 h immersion in citrate buffer (×5000). (**D**): SEM photomicrograph of IPS e.max ceramic after 168 h immersion in citrate buffer (×5000).

**Figure 7:**
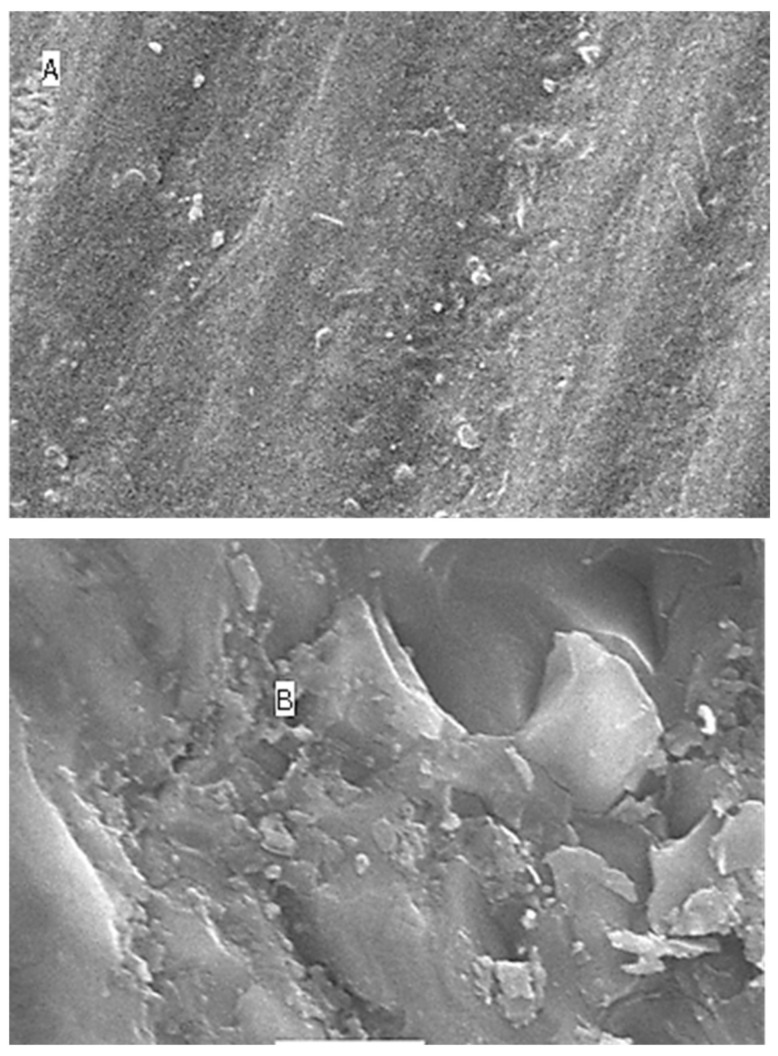

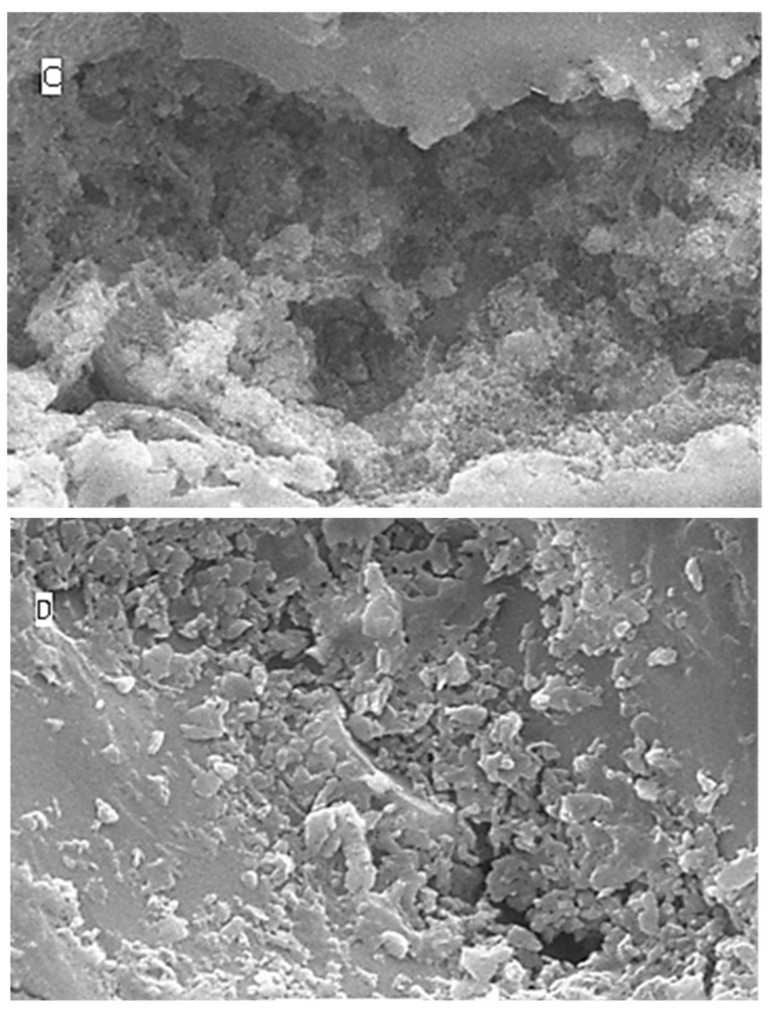
SEM images (**A**–**D**) of surface roughness at baseline, 24 h, 96 h and 168 h in 4% acetic acid. (**A**): Baseline SEM photomicrograph of IPS e.max ceramic before immersion in 4% acetic acid (×5000). (**B**): SEM photomicrograph of IPS e.max ceramic after 24 h immersion in 4% acetic acid (×5000). (**C**): SEM photomicrograph of IPS e.max ceramic after 96 h immersion in 4% acetic acid (×5000). (**D**): SEM photomicrograph of IPS e.max ceramic after 168 h immersion in 4% acetic acid (×5000).

**Figure 8:**
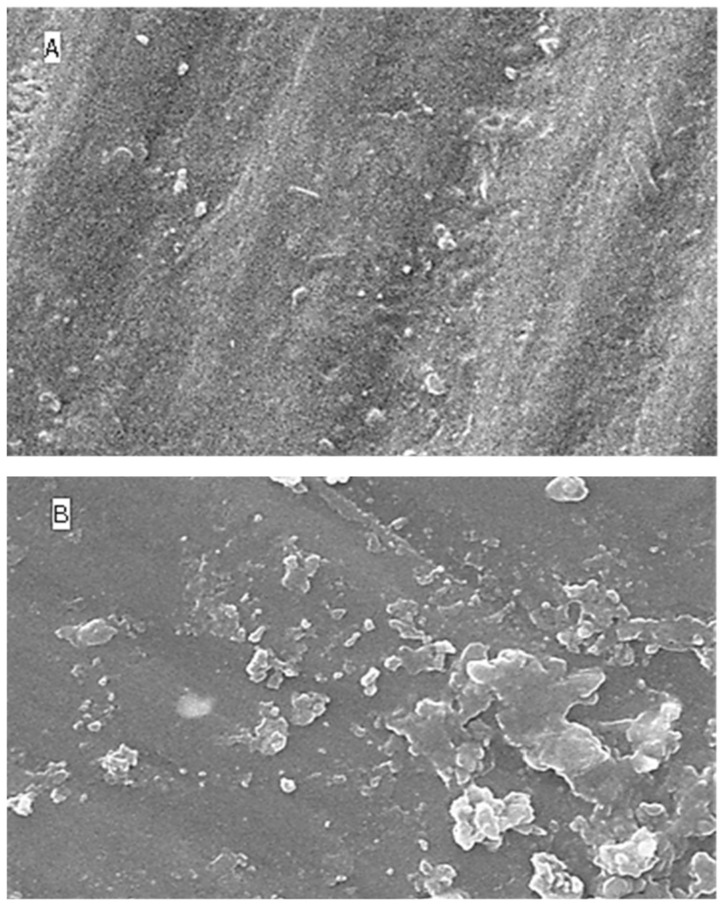

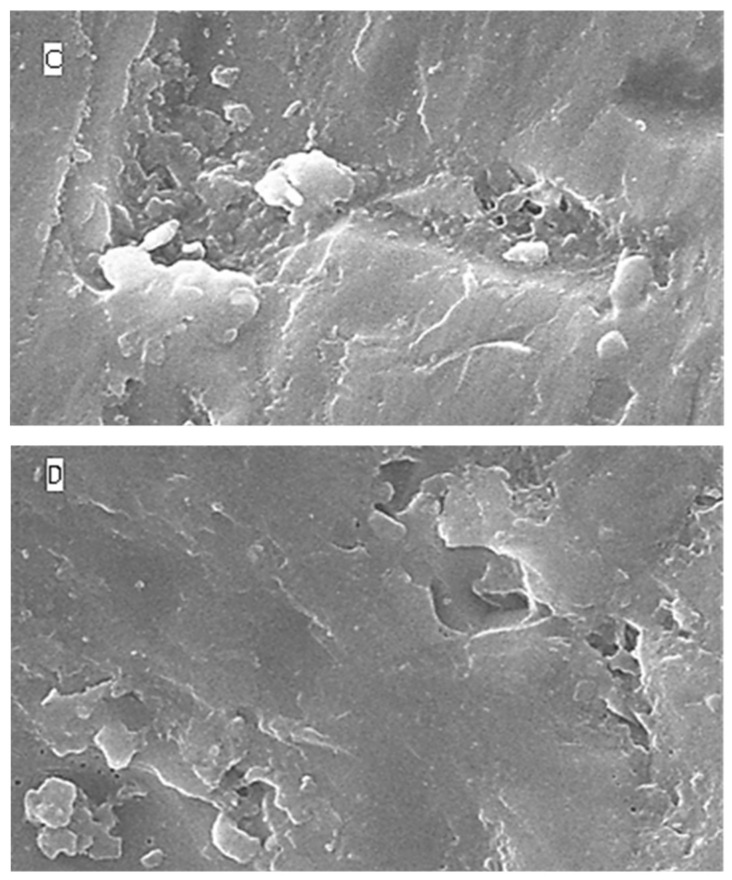
SEM images (**A**–**D**) of surface roughness at baseline, 24 h, 96 h and 168 h in soft cola. (**A**): Baseline SEM photomicrograph of IPS e.max ceramic before immersion in cola (×5000). (**B**): SEM photomicrograph of IPS e.max ceramic after 24 h immersion in cola (×5000). (**C**): SEM photomicrograph of IPS e.max ceramic after 96 h immersion in Cola (×5000). (**D**): SEM photomicrograph of IPS e.max ceramic after 168 h immersion in Cola (×5000).

The SEM results for surface roughness produced by the five different immersion media at four different time intervals are shown as Figure 4 (distilled water), Figure 5 (lemon juice), Figure 6 (citrate buffer), Figure 7 (acetic acid) and Figure 8 (soft cola), respectively. The SEM reports indicate a significant deterioration in the surface roughness of nano-fluorapatite ceramic when immersed in every immersion medium under consideration (lemon juice, citrate buffer, 4% acetic acid and cola) except distilled water. Prior to immersion in any medium, i.e., at baseline, the tested ceramic discs, according to the figures produced by SEM analysis showed a mostly smooth surface pattern with no noticeable changes on the ceramic disc surface.

In regard to distilled water group, for all time intervals (i.e., 24 h, 96 h, and 168 h), disc specimens did not show any specific surface degradation pattern, revealing a smooth surface with minor surface porosities and scratches (see Figure 4). These figures correspond with the profilometer outcomes that there was a minor (insignificant) increase in surface roughness upon prolonged immersion of disc specimens in distilled water.

The SEM microphotographs of ceramic disc surface after exposure to lemon juice revealed surface changes of varying degrees. Figure 5 illustrates that as the time interval increased from 24 h to 96 h and subsequently to 168 h; the surface irregularities and defects became larger and more pronounced. Similar results were reported for the citrate buffer group; Figure 6 reveals greater surface cracks and defects as the immersion time periodically increased, which showed the most surface deterioration at 168 h.

The greatest amount of surface irregularities and destruction were recorded for the 4% acetic acid testing group (see Figure 7) across all time intervals (i.e., at 24 h, 96 h, and 168 h), when compared with the other immersion groups. The SEM image for the soft cola group (see Figure 8) also showed larger surface defects at all time periods; however, they were of lesser severity in comparison to the acetic acid group.

## 3. Discussion

The use of ceramics in dentistry has become very popular because of their resistant nature. In ideal conditions, ceramic restorations should maintain their smooth and glazed surface finish during oral functions when they are routinely exposed to different erosive solutions and food substances. The surface of ceramics can be etched as a result of exposure to such acidic agents, thereby producing a roughened surface and compromised aesthetics [22]. Such roughened surfaces are prone to plaque accumulation, staining and further abrasion due to wear from opposing teeth [23]. Hence, these restorations require regular polishing to maintain their integrity.

It has been reported in the literature that metal–ceramic restorations have a clinical service life of up to 20 years [24,25]. The degradation process can be amplified by increasing the aggressiveness of testing agents in comparison to intraoral conditions. Hence, to exhibit the extensive effect of erosive solutions, a long immersion time interval i.e., 168 h was employed in the current study. It has been reported that 168 h exposure is equivalent to 22 years of immersion time in artificial saliva at 22 °C [25,26].

This study measured the pH of acidic agents (lemon juice, citrate buffer, 4% acetic acid and a cola drink) and investigated, quantitatively and qualitatively, the effects of distilled water, lemon juice, citrate buffer, 4% acetic acid and soft cola on the surface of nano-fluorapatite ceramic (IPS e.max) at baseline and after immersion time intervals of 24 h, 96 h and 168 h. The present research excluded the effects of wear produced by chewing habits, since it is extremely hard to simulate the complex natural intra-oral functions.

At present, in the field of dentistry, the two most commonly used methods to measure the surface roughness are a non-contact method that involves the use of a laser beam or a light beam to evaluate the profile of any surface. For surface roughness measurement, the most commonly used parameter is the Ra value [27], however the Ra value has a few limitations, including that it is a two-dimensional measure, thereby only providing data about the average surface height, and it does not give any information on the profile of the tested surface [27,28]. The SEM provides qualitative and subjective assessment of specimens. For this method, the specimens require gold-sputtering (coating) in order to produce a conductive layer for analysis [29,30].

Therefore, in order to overcome this limitation, scanning electron microscope (SEM) photomicrographs were utilized in the current study, which provides two-dimensional qualitative views. Hence, a combination of quantitative surface evaluation through Ra values and qualitative assessment by SEM analysis provides a true description of the evaluated specimen surfaces [31]. Additionally, measurements of surface roughness obtained from SEM scans may not represent or be generalized for the entire specimen surface. Hence, multiple measuring SEM analysis scans were taken in this study, which indicates that surface roughness values of ceramic disc specimens were in agreement with earlier literature findings [32].

In the current research study, a significant deterioration in the ceramic surfaces was observed in relation to all of the erosive media, which is in accordance with previous studies [1,5,15,19,33]. Hence, the study findings affirm that erosive agents produce roughness of the ceramic surfaces and, hence, the evaluated ceramic is not chemically inert or impermeable. The damage was quite prominent within 24 h of immersion and reached its peak at 168 h.

Although, insignificant (*p* < 0.829), very minor surface roughness or degradation was observed even in distilled water (a neutral aqueous solution), which was evident through the arithmetic Ra values of the profilometer and the SEM photomicrographs. A similar outcome was reported by Boonlert et al. [1]. and Junpoom et al. [19]. in their respective studies.

In an acidic environment, there is anion exchange between the protons present in erosive solutions and network modifiers in ceramic bulk. In order to understand the dissolution and subsequent degradation of ceramic, the process can be explained by two mechanisms; First, in ceramics there is a selective leaching of alkali ions, and second, dissolution of ceramic silicate network (Si–O–Si) occurs upon exposure to acidic solutions. These two processes are controlled by the exchange of ions between ceramic and the aqueous solution. The alkali ions diffuse from the ceramic surface into the immersed aqueous solution and there is reversible dispersion of hydrogen ions or hydronium ions (H_3_O^+^) from the aqueous solution into the ceramic to preserve electrical neutrality. Furthermore, it was observed by Upshaw et al. [34] that a low pH induced by exposure to acidic solutions resulted in a significant ion release from the e.max ceramic specimen discs. The ions included the primary network former (Si^4+^) as well as network modifiers (Ca, Zn, Li) present in the ceramic structure. In an acidic environment, there is anion exchange between the protons present in erosive solutions and network modifiers in ceramic bulk.

It is important to understand the two phases present in a ceramic, i.e., a crystalline phase and a glass phase. When a low pH solution (acidic in nature) comes in contact with ceramic, it attacks the glass phase, causing its breakdown and release of crystals into solution, which affects the kinetics of ion release and ultimately leads to dissolution and a roughened ceramic surface [33]. All testing agents (except distilled water) used in the present study are acidic in nature, having low pH values (citrate buffer, Coca-Cola, lemon juice and acetic acid) and have been shown to impart similar dissolution phenomenon. The present study confirms that exposure times at various pH levels are likely to lead to ceramic surface changes such as roughness and degradation.

However, in the present study the ion release from the ceramic structure in response to an acid attack was not investigated due to financial limitations; if performed, this would have demonstrated which essential ions are released when exposed to acidic solutions, thereby helping to understand the ceramic dissolution process and how ion leaching alters the mechanical and physical properties of a ceramic material.

The study findings support accepting the alternate hypothesis and rejecting the null hypothesis, since all the erosive agents except distilled water used as testing solutions produced significant surface roughness on the dental ceramic being tested. Acetic acid showed the highest amount of roughness followed by citrate buffer, cola and lemon juice, in decreasing order. Intra-oral ceramic restorations are regularly exposed to acid-base changes and temperature fluctuations produced by food and drink consumption. Therefore, in ideal circumstances the dental ceramic restorations should resist or only have minimal changes in such environments [35,36].

As per the results of roughness parameters and SEM photomicrographs of the present study, it is quite evident that 4% acetic acid was the most damaging immersion medium and produced a highly significant surface roughness of the evaluated ceramic in all dimensions (*p* < 0.001), when compared to the other groups. These surface roughness findings are in accordance with the observations of previous studies [1,5,12,15,19]. However, Boonlert et al. [16]. reported that fluorapatite crystals in IPS e.max Ceram are more durable as compared to previous feldspathic ceramic types.

Earlier studies have reported that surface roughness is inversely proportional to the strength of ceramics [37,38]. Hence, it is essential that ceramic restorations retain their strength and longevity despite continuous intra-oral exposure to regular acidic beverages. In context of the relationship between the state of the ceramic surface and oral hygiene, it has been reported that an arithmetic Ra value (denoting surface roughness) of 0.2 µm is critical for bacterial adhesion and colonization on the intra-oral restorative materials [32]. The baseline (before immersion) Ra value of the ceramic tested in this study is equivalent to the aforementioned critical value. In this study, the resultant increase in the surface roughness value of ceramic may lead to colonization of bacteria, a decline in strength and durability and, ultimately, clinical failure of the ceramic restorations. These observations highlight the fact that ceramic restorations require appropriate oral hygiene measures as well as regular professional maintenance to ensure that the surfaces of ceramic restoration are smooth and intact [39,40].

### 3.1. Study Strengths

- This is a study related to the performance of ceramics in clinical dentistry in local settings as it involves the utilization of a profilometer and scanning electron microscope (SEM) to record the changes which occur on the surface of ceramics when exposed to acidic beverages available on the local market.
- The present research focuses on IPS e.max Ceram, a nano-fluorapatite type ceramic, which is considered to be a superior ceramic in terms of its properties, therefore any surface effect on this high-quality type of ceramic gives an insight about the changes which can occur on other ceramic types.
- The research findings will provide clinicians and students an understanding of detrimental effects produced by routinely consumed acidic beverages and juices on ceramic restorations which are usually considered as inert restorative materials.
- The study findings will assist dental practitioners in disseminating useful information to their patients about caution relating to consumption of acidic beverages, thereby enhancing the longevity of their intra-oral ceramic restorations and reducing the chances of clinical failure.
- The current research provides dental practitioners with valuable information to plan dental restorations, keeping under consideration any underlying erosive conditions, such as GERD, bulimic conditions, alcoholism, and occupation-related exposures.

### 3.2. Study Limitations

- Since the present study was an in-vitro investigation, the influence of natural oral conditions such as the presence of saliva, water, intra-oral temperature changes and pH level could not be taken into consideration, since they may affect the study outcome. Henceforth, in vivo experiments should be undertaken to evaluate the true effect of erosive agents on dental ceramics.
- Due to limited financial resources, only one ceramic type i.e., fluorapatite (IPS e.max Ceram), was evaluated in the current study. Therefore, more studies are required to determine the impact of acidic beverages/agents on other types of dental ceramics.
- Artificial saliva could have been used as an additional test group, since it offers a better medium to reproduce natural oral conditions.
- A contact-type profilometer was used in the present study. A disadvantage of this type is that the stylus tip, upon contact with disc specimen, causes irreversible damage to the discs. Hence, such specimens cannot be re-used for further study purposes.

### 3.3. Recommendations

- The effect of natural saliva on the ceramic surface should be explored to determine its behavior in in vivo conditions.
- Additional potential erosive juices and beverages should be included to explore their effect on ceramics.
- More ceramic types should be added in future research endeavors.
- Additional testing methods, including atomic force microscopy (AFM) should be employed to give a three-dimensional image of the ceramic surface.
- The effect of temperature changes should be included in future studies.
- Titrability of acidic drinks, along with their respective pH values, should be considered in future endeavors.
- The synergistic effect of abrasion/attrition on wear with chemical erosion should be assessed in further studies.

## 4. Materials and Methods

### 4.1. Specimen Fabrication

The dental ceramic selected for the study was IPS e.max Ceram (Ivoclor Vivadent, Amherst, NY, USA) A total of 160 disc specimens (12 mm diameter and 2 mm thickness) were made using a prefabricated stainless-steel mold. For disc preparation, ceramic powder was mixed with deionized water using a plastic spatula on a glass slab and the powder/liquid ratio was maintained according to the manufacturer’s instructions. The creamy mix was condensed into a metal mold and covered with a platinum foil and glass slab. After condensation, excess liquid on the surface of specimens was blot-dried with absorbent paper and they were lifted from their molds and kept on the platinum foil before the sintering process. The test specimen discs were cured in a chamber/vacuum furnace as per the manufacturer’s instructions. Following sintering, the specimens were polished using a rubber disc. Finally, the specimens were subjected to self-glazing treatment as per the manufacturer’s instructions.

### 4.2. Solution Preparation and pH Testing

Four types of erosive agents were used in this experimental study (Table 5), namely: lemon juice, citrate buffer solution, 4% acetic acid and cola drink. The pH value of 25 mL of each erosive agent was measured three times to provide a mean pH value of each agent to be used. The pH of all solutions was maintained at all time intervals.

### 4.3. Specimen Immersion and Surface Characteristic Changes Using Profilometer and SEM

One hundred and sixty ceramic specimen discs were evenly divided into two groups (*n* = 80) to assess the surface roughness and surface topographical changes respectively. The baseline reading of five specimens for each of the two groups was recorded. The profilometer testing group (*n* = 80) was further divided into five sub-groups (*n* = 15) to be exposed to distilled water (control) and four erosive agents. The discs for the acetic acid group were immersed in 4% acetic acid at 80 °C (ISO 6872) [41] using a hot air oven (Binder, Tuttlingen, Germany) and then tested for surface roughness at time intervals of 24 h, 96 h and 168 h respectively. For the remaining immersion groups, 60 (15 × 4) polythene bottles were used, with each bottle containing single porcelain disc and 25 mL of testing solution, and were sealed with a screw cap. The discs were stored in an incubator, having an optimum temperature of 37 °C and were then tested three times at 24 h, 96 h, and 168 h intervals. The surface roughness was recorded as ‘Ra’ values [42,43,44,45] (arithmetical average of surface heights in µm) and these Ra values were measured at five different positions (at least 1.5 mm apart) on the disc specimen with a force of 4 mN, stylus speed of 0.5 mm/s and a cutoff value of 0.8 mm, and the mean values were recorded. The solutions were discarded after each test.

A similar protocol of immersion was employed for the SEM group; however, prior to SEM analysis, the specimens were rinsed with distilled water for 5 min and dried, followed by sputter-coating with gold-palladium alloy (SPI-Module sputter, SPI Supplies, West Chester, PA, USA) for 8 min, resulting in a film thickness of about 100–300 nm, and examined under the SEM.

### 4.4. Data Management and Analysis

The data were analyzed with Statistical Package for the Social Sciences SPSS-25). (SPSS Inc., Chicago, IL, USA). Descriptive analysis was performed to compute mean and standard deviation values of the surface roughness of dental ceramic when exposed to different erosive agents. ANOVA with post hoc analysis (Tukey’s HSD test) was applied to compare surface roughness parameters among the testing solution groups. Repeated measures ANOVA was executed to compare the baseline value with each immersion time interval. A *p*-value of ≤0.05 was considered as significant.

## 5. Conclusions

The conclusions drawn from the current study are as follows:

- The tested erosive agents had a negative effect on the surface roughness of tested ceramic.
- A significant deterioration in the ceramic surfaces was observed in relation to all of the immersion media except distilled water.
- The surface roughness increased with increased storage time interval.
- The low pH found in beverages and fruit juices was observed to be a causative factor in increasing the surface roughness of the material.
- This study outcome should be considered during advertising of and in consumers’ habits of consuming erosive beverages.

## Figures and Tables

**Table 1:** Profilometer readings distribution for nano-fluorapatite ceramic immersed in distilled water at baseline and for 24 h, 96 h, and 168 h.

Specimen	Baseline Mean, SD	24 h Mean, SD	96 h Mean, SD	168 h Mean, SD
Nano-fluorapatite ceramic	0.223 ± 0.004	0.224 ± 0.005	0.224 ± 0.005	0.224 ± 0.005

**Table 2:** Profilometer readings distribution for nano-fluorapatite ceramic immersed in citrate buffer at baseline and for 24 h, 96 h, and 168 h.

Specimen	Baseline Mean, SD	24 h Mean, SD	96 h Mean, SD	168 h Mean, SD
Nano-fluorapatite ceramic	0.223 ± 0.004	0.274 ± 0.005	0.325 ± 0.005	0.366 ± 0.006

**Table 3:** Comparison of surface roughness (mean Ra value) of nano-fluorapatite ceramic across four different time intervals for each immersion solution.

Storage Agent	Baseline Mean, SD	24 h Mean, SD	96 h Mean, SD	168 h Mean, SD	*p*-Value
Distilled Water	0.223 (±0.004)	0.224 (±0.005)	0.224 (±0.005)	0.224 (±0.005)	0.8289
Lemon Juice	0.223 (±0.004)	0.260 (±0.008)	0.286 (±0.004)	0.349 (±0.005)	<0.0001 ^a^
Citrate Buffer	0.223 (±0.004)	0.274 (±0.005)	0.325 (±0.005)	0.366 (±0.006)	<0.0001 ^a^
Acetic Acid	0.223 (±0.004)	0.293 (±0.004)	0.370 (±0.007)	0.421 (±0.007)	<0.0001 ^a^
Soft Cola	0.223 (±0.004)	0.245 (±0.007)	0.290 (±0.007)	0.355 (±0.007)	<0.0001 ^a^

^a^: *p*-values were calculated with a post-hoc comparison using Tukey’s HSD, showing there was a statistically significant difference between mean Ra values at baseline and 24 h, at 24 h, and 96 h, as well as at 96 h, and 168 h, when ceramic discs were immersed in lemon juice, citrate buffer, acetic acid, and soft cola.

**Table 4:** Comparison of surface roughness (mean Ra value) of nano-fluorapatite ceramic between five immersion media at different time intervals.

Time (Hours)	Distilled Water Mean, SD	Lemon Juice Mean, SD	Citrate Buffer Mean, SD	Acetic Acid Mean, SD	Soft Cola Mean, SD	*p*-Value
Baseline	0.223 (±0.0048)	0.223 (±0.004)	0.223 (±0.004)	0.223 (±0.004)	0.223 (±0.004)	1.00
24 h	0.224 (±0.0051)	0.260 (±0.008)	0.274 (±0.005)	0.293 (±0.009)	0.245 (±0.001)	<0.001 ^a^
96 h	0.221 (±0.0052)	0.286 (±0.004)	0.325 (±0.003)	0.370 (±0.007)	0.290 (±0.004)	<0.001 ^a^
168 h	0.225 (±0.0057)	0.344 (±0.005)	0.366 (±0.006)	0.421 (±0.005)	0.355 (±0.007)	<0.001 ^a^

^a^: *p*-values were calculated with post-hoc comparison using Tukey’s HSD, showing there was statistically significant difference between mean Ra values at baseline and 24 h, at 24 h and 96 h as well as at 96 h and 168 h when ceramic discs were immersed in lemon juice, citrate buffer, acetic acid and cola.

**Table 5:** Details of type, preparation, composition, measured pH and manufacturer of the immersion media used for the study.

Erosive Media	Prepared/Form	Composition	pH Mean and SD	Manufacturer
Lemon juice	Prepared from fresh lemons	Lemon juice vol%	2.21 ± 0.03	Laboratory prepared
Citrate buffer solution	Instant	Mixture of 5-chloro-2-methyl-4-isothaizolin-3-one and 2-methyl-4-isothaizolin-3-one (3:1)	4.99 ± 0.01	Laboratory prepared
Acetic acid	Diluted from 100% acetic acid	4% acetic acid	2.47 (0.01)	Laboratory prepared
Soft Cola drink	Instant	Mixture of sugar, water, lime juice, citrate of caffeine, citric acid, extract of vanilla, fluid extract of kola nut and fluid extract of coca	2.41 (0.06)	Coca Cola™ Company

## Data Availability

The data presented in this study are available on request from the corresponding author.

## Sample Availability

Samples of the compounds are available from the authors.
